# Advanced Computational Methods for Modeling, Prediction and Optimization—A Review

**DOI:** 10.3390/ma17143521

**Published:** 2024-07-16

**Authors:** Jaroslaw Krzywanski, Marcin Sosnowski, Karolina Grabowska, Anna Zylka, Lukasz Lasek, Agnieszka Kijo-Kleczkowska

**Affiliations:** 1 Department of Advanced Computational Methods, Faculty of Science and Technology, Jan Dlugosz University in Czestochowa, Armii Krajowej 13/15, 42-200 Czestochowa, Poland; m.sosnowski@ujd.edu.pl (M.S.); k.grabowska@ujd.edu.pl (K.G.); a.zylka@ujd.edu.pl (A.Z.); 2 Wladyslaw Bieganski Collegium Medicum, Jan Dlugosz University in Czestochowa, Armii Krajowej 13/15, 42-200 Czestochowa, Poland; l.lasek@ujd.edu.pl; 3 Department of Thermal Machinery, Faculty of Mechanical Engineering and Computer Science, Czestochowa University of Technology, 42-201 Czestochowa, Poland; a.kijo-kleczkowska@pcz.pl

**Keywords:** artificial intelligence, machine learning, modeling, simulation, optimization, complex systems, material engineering, energy systems

## Abstract

This paper provides a comprehensive review of recent advancements in computational methods for modeling, simulation, and optimization of complex systems in materials engineering, mechanical engineering, and energy systems. We identified key trends and highlighted the integration of artificial intelligence (AI) with traditional computational methods. Some of the cited works were previously published within the topic: “Computational Methods: Modeling, Simulations, and Optimization of Complex Systems”; thus, this article compiles the latest reports from this field. The work presents various contemporary applications of advanced computational algorithms, including AI methods. It also introduces proposals for novel strategies in materials production and optimization methods within the energy systems domain. It is essential to optimize the properties of materials used in energy. Our findings demonstrate significant improvements in accuracy and efficiency, offering valuable insights for researchers and practitioners. This review contributes to the field by synthesizing state-of-the-art developments and suggesting directions for future research, underscoring the critical role of these methods in advancing engineering and technological solutions.

## 1. Introduction

In recent years, an intense increase in interest in artificial intelligence and computational methods has been observed. Their main goal is to create and identify systems incorporating or exhibiting intelligence, including human and animal behavior. They connect with other fields, e.g., mathematics, neuroscience, epistemology, mental health studies, and language studies, as well as informatics. Hence, artificial intelligence (AI), including machine learning (ML) are increasingly wide applications for complex systems.

Since this paper reviews recent developments in artificial intelligence and computational methods focusing on the modeling, simulations, and optimization of complex systems in materials science, we should start by discussing emerging trends in AI, as now we can conduct virtual simulations that provide us with a depiction of the information landscape based on current knowledge.

In their article, Gill et al. [1] presented the latest studies and possible future trends in cloud computing, AI/ML, and quantum computing. They further explored the hurdles and possibilities in utilizing AI, including ML, within advanced computing frameworks, such as fog, edge, serverless, and quantum environments, as well as cloud computing architectures. In paper [2], the authors reviewed fog computing and machine learning, covering theories, practical uses, obstacles, and unresolved questions.

An innovative method for creating neuromorphic electronic devices with extremely low energy usageis provided in [3]. The authors highlighted that these devices can replicate typical biological synaptic functions, such as excitatory postsynaptic currents, short- and long-term plasticity, and the process of “learning”, using vertical organic field-effect transistor-based optoelectronic synaptic devices.

Artificial intelligence can also be used to design electromagnetic materials. In paper [4], the authors presented, among other things, the potential applications of negative permittivity materials (considered a supernormal property) in dielectric capacitors and electromagnetic shielding. The authors of article [4] offered guidance for fabricating flexible materials with negative permittivity.

They presented results of preparing the flexible cementite/ferro ferric oxide/silicon dioxide and carbon nanofibers, exhibiting weak and low-frequency dispersion with negative permittivity, fabricated via electrospinning and high-temperature carbonization. Jiaqi Jiang et al. [5] discuss utilizing convolutional neural networks and recurrent neural networks in photonic devices. They pointed out that trained deep neural networks can serve as high-speed surrogate electromagnetic solvers.

A review of various AI-based techniques utilized in intestinal endoscopy to detect colonic polyps can be found in [6]. The authors stated that AI-based platforms have achieved clinically acceptable diagnostic efficiency in the automated diagnosis of polyps.

In the paper [7], Svítek explored the expanded concept of information systems, incorporating pure quantum principles utilizing wave probability functions analogous to electrical circuits and quantum physics. The key parameter addressed was the management of information flux and information richness, ensuring equilibrium. According to Tao et al. [8], the effectiveness of feature extraction is crucial in speech emotion recognition. The authors introduced a multi-stream convolutional recurrent neural network utilizing the attention mechanism (MSCRNN-A). It enabled the extraction of discriminative, affect-salient features from speech signals and improved speech emotion recognition.

Jonathan Schmid et al. [9] discussed ML algorithms for crystal structure prediction. The authors underlined the high potential of integrated ML techniques with other numerical methods, including molecular dynamics and global structural prediction, for material science tasks. Valentin Stanev et al. [10] discussed AI models used in quantum materials. Moreover, they proposed a real-time closed-loop idea for autonomous systems in materials research and optimization.

**Figure 1:**
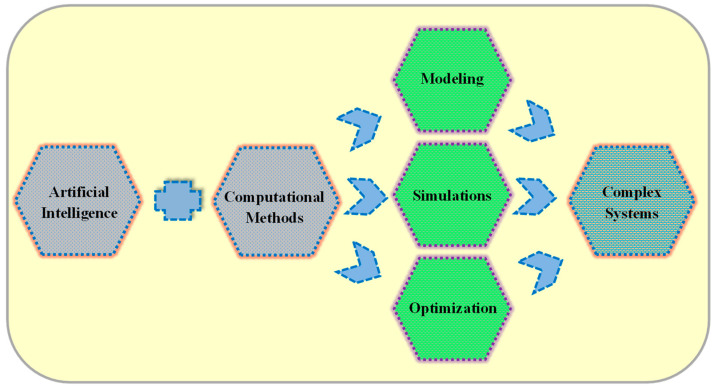
Scheme of approach utilizing a combination of artificial intelligence and computational methods for complex system modeling, optimization, and simulations.

The dynamic development of artificial intelligence and other computational methods makes it necessary to organize the latest achievements in this paper. The review seeks to integrate research on advancements in complex systems modeling, simulations, and optimization challenges. It collects current and timely papers on artificial intelligence and other computational methods. The paper considers optimization techniques and algorithms, new strategies in advanced computing, and AI approaches, providing potential research directions in different fields, especially in materials engineering, presented in detail in the following chapters of the manuscript (Figure 1).

Therefore, the review aims to synthesize state-of-the-art developments in computational methods, identify the most effective algorithms for specific applications, and advocate for integrating AI with traditional methods to accelerate scientific and technological progress.

## 2. Methodology

As modern data processing systems gain greater computational power, new possibilities arise for the modeling, simulation, and optimization of complex systems and devices. In various scientific and technological fields, comprehensive and advanced models can be developed using challenging, resource-intensive, and time-consuming techniques. By integrating AI algorithms with computational strategies, including numerical and alternative methods, it becomes feasible to perform parallel analyses and address sophisticated and interdisciplinary issues.

The papers discussed in this review are selected based on the performance and effectiveness of the methods and approaches considered, providing a better focus on specific problems that can be useful for readers.

The central axis of the paper is the broad research area of papers published on the Topic “Artificial Intelligence and Computational Methods: Modeling, Simulations, and Optimization of Complex Systems”, which covers 74 published papers.

To ensure a robust and systematic review, we employed a structured literature search strategy. We searched several reputable databases, including IEEE Xplore, ScienceDirect, SpringerLink, and Google Scholar. Our search utilized specific keywords such as “artificial intelligence, “machine learning”, “modelling”, “simulation”, “optimization”, “complex systems”, “material engineering”, “energy systems”. Using the aforementioned keywords and databases, the most relevant paper were identified. These articles were screened based on their relevance to our research objective, narrowing the selection. A detailed review of the selected papers was conducted, focusing on their methodologies, findings, and contributions to the field.

Such a vast amount of material resulted in a wide range of topics discussed, which is an absolute value of the presented review.

## 3. Advanced Computational Methods for System Analysis and Prediction

Advanced computational techniques are divided into several categories, including classification techniques, which are not always independent of one another and can be used not only in the classification tasks. Some of them are listed throughout the paper. However, it is important to emphasize that each category is characterized by a different methodological approach.

Machine learning (ML) is considered a data-driven approach. ML algorithms are designed to learn patterns and make predictions based on data. On the other hand, numerical simulation term is encompassed within the broader category of mathematical modeling and numerical methods. Mathematical modeling involves the formulation of mathematical models of real-world systems or processes, while numerical methods refer to a range of computational techniques used to approximate solutions to these mathematical models, especially when analytical solutions are intractable.

Computational methods for system analysis and prediction refer to sophisticated techniques and approaches used in computational science to analyze complex systems and make predictions based on the available data. These methods often involve using advanced mathematical models, algorithms, and computational tools. They are used to simulate, explore, and predict the behavior of intricate systems in various domains, such as material science, engineering, physics, biology, and finance.

Key aspects and techniques of advanced computational techniques for system analysis and prediction are as follows:

- Mathematical modeling and numerical methods: This includes using differential equations to describe various systems and employing numerical methods to solve these equations numerically. For example, agent-based modeling represents individual entities (agents) and their interactions to simulate the entire system’s behavior [11,12].
- Numerical simulation: Techniques such as Finite Element Analysis (FEA) are widely used in engineering tasks to analyze and predict the behavior of structures and materials under various conditions. Similarly, Computational Fluid Dynamics (CFD) is applied to simulate the flow of fluids and predict their interaction with solid boundaries [13,14,15].
- Machine learning and artificial intelligence: These algorithms are trained on historical data to predict future events or trends. Machine learning can be seen as both a data-driven approach and an optimization technique, and it is also included under the broader umbrella of artificial intelligence. Deep learning models, such as neural networks, are used for complex pattern recognition and prediction tasks, forming a subset of artificial intelligence [16,17,18,19].
- Optimization techniques: These often use genetic algorithms inspired by natural selection to solve optimization problems where the search space is very extensive [20].
- Data-driven approaches: Techniques like big data analytics are used for analyzing large datasets to extract meaningful patterns and trends for prediction purposes. Data assimilation integrates observational data into models to improve their accuracy over time [21,22].
- Complex systems theory: This includes the study of nonlinear dynamics, which investigates how dynamic systems respond to initial states with high sensitivity, leading to seemingly random results. Network theory, used for analyzing the interactions and dependencies among components in a system represented as a network, is another branch of complex systems theory. These principles are crucial in mechanical engineering applications, such as the development of soft sensors for predicting temperature fields in rotary kilns and the implementation of image recognition systems for temperature control in industrial processes [23,24].
- Uncertainty quantification: Methods such as Monte Carlo simulation generate random samples to estimate the probability distribution of uncertain parameters, while Bayesian methods incorporate prior knowledge and update predictions based on new data [25,26].
- High-performance computing (HPC): This leverages the power of supercomputers and parallel processing to handle large-scale simulations and analyses [27,28,29].

Some very interesting examples of research using different techniques of advanced computational methods for system analysis and prediction are presented in the following paragraphs. They include an extensive learning framework utilizing the sparrow search algorithm, a hybrid-flash butterfly optimization algorithm, an attention-based isolation forest approach, and others.

### 3.1. The Sparrow Search Algorithm

An analysis of predicting network interface flow utilizing a comprehensive learning system based on the sparrow search algorithm was conducted, as discussed in [30]. In addition to various metrics used for assessment, a moving average (1—mean absolute percentage error) was also employed as an additional evaluation indicator. The smaller the first four errors and the higher the moving error, the better. This means that while the mean squared error, the root mean squared error, the mean absolute error, and the mean absolute percentage error are closer to zero, and the moving error is closer to 100%, it signifies enhanced model prediction accuracy.

To streamline the intricate manual parameter tuning process and achieve optimal combinations of hyperparameters, the authors utilized novel search techniques to fine-tune the shrinkage coefficient (r) and regularisation coefficient (λ) in the expansive learning framework. This aimed to improve the model’s predictive precision. Subsequently, a model was constructed using a broad approach to learning for predicting traffic patterns. Specifically, the traffic flow values from the interval [t − 1, t] were utilized as key indicators for predicting traffic at time t + 1. The parameters obtained earlier were integrated into the network for training the prediction model for network traffic. Finally, model performance was evaluated through training on two publicly available network flow datasets and precise traffic data from an enterprise cloud platform switch interface. A comparative analysis was performed across different methodologies, including long short-term memory, among others. The authors of [30] presented the experimental findings from a core network traffic dataset in European cities in Table 1. SSA-BLS and SCN demonstrated the lowest MSE and RMSE values among the models evaluated, signifying superior prediction accuracy. The SSA-BLS model, in particular, achieved an MSE of 0.0159 and an RMSE of 0.1261, outperforming other models like ELM and LSTM, which had considerably higher error rates. Furthermore, SSA-BLS sustained a high mean accuracy of 97.06%, highlighting its effectiveness in predicting network traffic. Traditional models, such as ELM and LSTM, perform inferior to more advanced models like SSA-BLS and SCN.

The experiments revealed that this approach demonstrated higher prediction accuracy than alternative methods, achieving moving averages of 97% for a primary urban network traffic dataset (Table 1), 98% for a dataset from the UK academic backbone network, and 99% for traffic observed on an enterprise cloud platform switch interface.

### 3.2. Hybrid-Flash Butterfly Optimization Algorithm

An interesting optimization approach was described in [31], employing a hybrid-flash butterfly optimization algorithm (HFBOA) tailored for addressing engineering-constrained optimization challenges. The butterfly optimization algorithm (BOA) traditionally focuses solely on the smell perception rule, rendering it susceptible to local optimum traps. In contrast, the HFBOA introduces an additional operator, namely the color perception rule, aiming to align the algorithm with the natural foraging behaviors of butterflies. Furthermore, the HFBOA incorporated an updated strategy for control parameter adjustment through logistic mapping, enhancing its global optimization capabilities. The method’s efficiency was evaluated using twelve benchmark functions, with comparative experiments indicating that the HFBOA exhibited faster convergence and greater stability in addressing numerical optimization challenges compared to other advanced optimization techniques. Moreover, the HFBOA successfully addressed a variety of engineering optimization tasks. Simulation results confirmed its effectiveness in solving complex real-world engineering challenges.

### 3.3. Curriculum Reinforcement Learning

With the ongoing advancement of reinforcement learning techniques in intelligent systems, incorporating adaptive learning strategies could potentially boost algorithmic effectiveness and optimize learning processes. This integration is particularly beneficial across varying difficulty levels. Many existing automatic curriculum learning algorithms rely on expert experience and a single network for curriculum ranking, leading to challenges in accurately ranking curriculum tasks and achieving swift convergence. Therefore, an examination of reinforcement learning methodologies utilizing robust validation techniques was presented in [32], addressing advanced computational methods for system analysis. The authors introduce a curriculum reinforcement learning technique to estimate task curricula’ relative difficulty scores. Drawing inspiration from the human notion of advancing from simple to complex in educational learning, the technique splits automated educational progression into two phases: an educational challenge evaluation phase and an educational arrangement phase. This strategy enables enhanced ordering of educational tasks through concurrent instruction of the teaching model and mutual assessment of the complexity of task examples. The reinforcement learning method based on automated curriculum learning offers the benefits of rapid solution speed and robust model generalization, making it particularly effective for addressing optimization problems in industrial settings. Additionally, it can introduce new strategies and concepts for tackling combinatorial optimization challenges. In conclusion, using automatic curriculum learning with K-Fold cross-validation improves the training speed of the Multi-Agent Deep Deterministic Policy Gradient (MADDPG) algorithm. Furthermore, it demonstrated a degree of generalizability for multi-agent deep reinforcement learning algorithms that utilize the replay buffer mechanism.

### 3.4. Attention-Based Isolation Forrest

The application of advanced computational methods for anomaly prediction was effectively investigated in [33]. The authors introduced a novel adaptation of the isolation forest as the Attention-Based Isolation Forest (ABIForest) to address the anomaly detection challenge. This modification integrated an attention mechanism, specifically the Nadaraya–Watson regression, into the isolation forest framework to enhance anomaly detection solutions. The core concept behind this enhancement involved assigning attention weights to individual paths within the tree structure. Using Huber’s contamination model was recommended for specifying these focus magnitudes and their associated characteristics. Subsequently, the focus magnitudes rely linearly on adjustable focus characteristics, optimized through resolution of a conventional linear or quadratic optimization issue. Notably, ABIForest represented the initial modification of the isolation forest that straightforwardly incorporates an attention mechanism without resorting to gradient-based algorithms.

### 3.5. Clustering-Based Redundancy Identification

Wu et al. used a different approach [34]. The authors adopted a distinct strategy, suggesting model pruning based on structural redundancy. They argued that recognizing functionally similar filters is crucial and introduced a model pruning centers on clustering-based redundancy detection. A corresponding iterative pruning scheme was proposed to address the issue of prolonged compression periods resulting from excessive fine-tuning. Thorough testing confirmed the efficacy of their compression approach. Detailed evaluations across various network architectures and datasets demonstrate the robustness of the framework put forward by the authors.

The above-described applications of computational algorithms and AI methods primarily motivate the expansion of practical implementation areas of these advanced tools. Such areas could include the management of geothermal energy systems or the modeling of chaotic processes, which are addressed in the following sections of the review paper.

### 3.6. The Gradient-Boosted Regression Tree for Geothermal Heat Flow

A very interesting prediction analysis concerning terrestrial heat flow using a machine learning method was featured in [35], leveraging a variety of geological and geophysical data sources. Geothermal heat flow is a crucial parameter in exploring geothermal energy. The cost often becomes a barrier when obtaining dense heat flow measurements across a study area. Nevertheless, expanding the few and scattered measurements of heat flow is imperative for understanding the regional geothermal landscape. Therefore, the research holds significance in generating a dependable map of terrestrial heat flow, laying the groundwork for future geothermal resource development. The gradient-boosted regression Tree (GBRT) prediction model was employed to address the challenge of insufficient heat flow observations. The model incorporated 12 geological and geophysical features to train sample data, thereby considering the geological and geophysical characteristics of the region. The result of the carried-out research was a robust GBRT prediction model. Performance evaluation involved comparing the GBRT method with kriging interpolation, as well as other interpolation techniques, through an analysis of prediction performance. The authors randomly separated 379 geodetic heat flow samples from the North China Craton, utilizing 80% for training and the remaining 20% for validation, and applied the previously mentioned methods for prediction. Subsequently, the outcomes were evaluated against the observed values in the validation set. The linear correlation analysis between the validation set’s heat flow measurements and the predicted values from the four methods showed that the average absolute errors for the GBRT, the kriging interpolation, the minimum curvature interpolation, and the 3D interpolation methods were 10.10, 10.59, 11.01 and 12.52, respectively. The lowest error characterizes the GBRT method, and the 3D interpolation gains the highest error of all the analyzed methods. The normalized mean square error was 0.22, 0.23, 0.24, and 0.30, respectively, indicating similar error values behavior for the analyzed methods. Therefore, the GBRT exhibited the highest accuracy among the four methods, whereas the 3D interpolation demonstrated the lowest. The new prediction model produced a heat flow map at a 0.25° × 0.25° resolution, offering a more detailed and accurate depiction of terrestrial heat flow distribution compared to interpolation results. Its reliability in obtaining precise results in areas with limited and irregularly distributed heat flow observations was proven, contributing to a more comprehensive understanding of geothermal conditions.

### 3.7. Support Vector Methods

Incorporating artificial intelligence into mechanical materials marks a pioneering approach that brings about a revolutionary shift in the predictive modeling process. This integration signifies a technological advancement and heralds a transformative paradigm, fundamentally reshaping the landscape of predictive modeling in mechanical materials. By leveraging the potential of artificial intelligence, specifically through machine learning algorithms like linear regression (LIR) and support vector regression (SVR), it becomes possible to discern intricate relationships among a diverse range of variables precisely. This technological advancement enables precise prediction of mechanical properties, thereby optimizing the engineering material design process [36]. This approach was employed in the work by Ward et al. [37]. Their methodology utilizes a chemically diverse set of attributes, convincingly illustrated to be well-suited for describing a broad spectrum of properties. Furthermore, they introduce an innovative approach to partitioning the dataset into clusters of similar materials, thereby enhancing predictive accuracy. This dual-pronged strategy highlights the versatility of the chosen attributes in capturing diverse properties. It underscores the significance of their novel data partitioning method in improving the precision of predictions for a wide range of materials. Progress in AI-based methods, data acquisition, and management techniques manifests in many ways. For example, Li and Liu [38] proposed a predictive strategy that guides investment and decision-making processes based on time-series data analysis. The authors concluded that the support-vector-machine-based (SVM) method provided the best prediction performance using easily accessible data. Additional values of the proposed approach include its capacity to impact future developments and its ability to shed light on market trends.

### 3.8. Solution for Modeling Chaotic Behavior

Many researchers apply machine learning to predict chaotic time series, as it has posed a persistent challenge in recent decades. A very interesting research in this area is presented in [39], where a hybrid approach has been devised, integrating the Hankel Alternative View Of Koopman (HAVOK) analysis with machine learning (HAVOK-ML) to enhance the accuracy of chaotic time series predictions. HAVOK-ML employs a closed linear model reconstruction to simulate the time series, effectively facilitating the prediction process. Through HAVOK analysis, chaotic dynamics are deconstructed into intermittently forced linear systems, and machine learning is utilized to estimate the external intermittently forcing term. Evaluations of prediction performance affirm that the proposed method exhibits superior forecasting capabilities compared to other prediction methods. Hu and Mao [40] introduced the Global Recurrence Plot (GRP)-based Generative Adversarial Network (GAN) and the Long Short-Term Memory (LSTM) combination method, named GRP-LSTM-GAN, for forecasting rotary kiln temperatures. They developed a GRP model to convert 1D chaotic time series into 2D images, capturing global and local features. This resulted in the creation of a GRP-LSTM-GAN prediction model that outperforms existing methods in terms of prediction accuracy. The comparison of methods is given in Table 2.

The experiments demonstrated that this approach enhances the convergence speed of the model and generates more lifelike GRPs. Furthermore, relationship between the input variables was examined. The strongest link with coal usage occurred with a 178-unit delay in the temperature data. This indicates that the model achieved its best predictive accuracy for temperature with a 178-time unit lag, using both coal usage and temperature information. Sintering temperature (ST) in rotary kilns is a fundamental system parameter, playing a pivotal and widespread role in controlling the sintering process. It significantly influences production quality and energy consumption, making it a crucial factor in sintering technology. Consequently, investigating sintering temperature holds great value for industrial applications [46].

**Figure 2:**
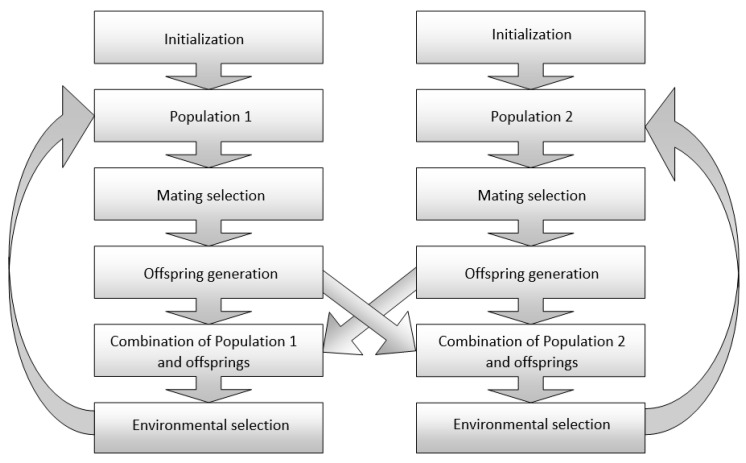
Simplified diagram of the DP-NSGA-III process.

Geng et al. [47] employed a dual-population-based evolutionary algorithm designed to handle constrained many-objective problems, known as DP-NSGA-III. Compared to existing Constrained Many-Objective Evolutionary Algorithms (CMaOEAs) [48], DP-NSGA-III evolves through the exchange of offspring between two populations. The primary obstacle in dealing with Constrained Many-Objective Optimization Problems (CMaOPs) lies in finding a balance between feasible and infeasible solutions. An ε-constraint handling approach was devised with NSGA-III, aiming to leverage the strengths of excellent infeasible solutions within the primary population. The method is graphically presented in Figure 2. The authors demonstrated the effectiveness of the suggested algorithm in efficiently resolving CMaOPs.

Enhancing materials’ strength and energy absorption properties is crucial to developing materials technology [49]. The introduction of the pelican optimization algorithm (POA) has been refined by Mei et al. [50], who utilized the Latin hypercube sampling (LHS) method and the Chaotic mapping (CM) techniques to enhance the random forest (RF) model for forecasting the seismic properties of an innovative seismic rubber-concrete material. The findings from the conducted research suggest that predicting strength is remarkably accurate for rubber, whereas cement proves to be a reliable material when forecasting energy absorption properties. This study indicates that using intelligent models for assessing the seismic performance of materials represents a practical and simplified methodology.

These achievements in using artificial intelligence and computational algorithms to optimize, model, and predict complex systems confirm their application potential. The further increase in available computer computing power encourages the search for new computational algorithms, such as gene expression programming.

### 3.9. Gene Expression Programming

**Figure 3:**
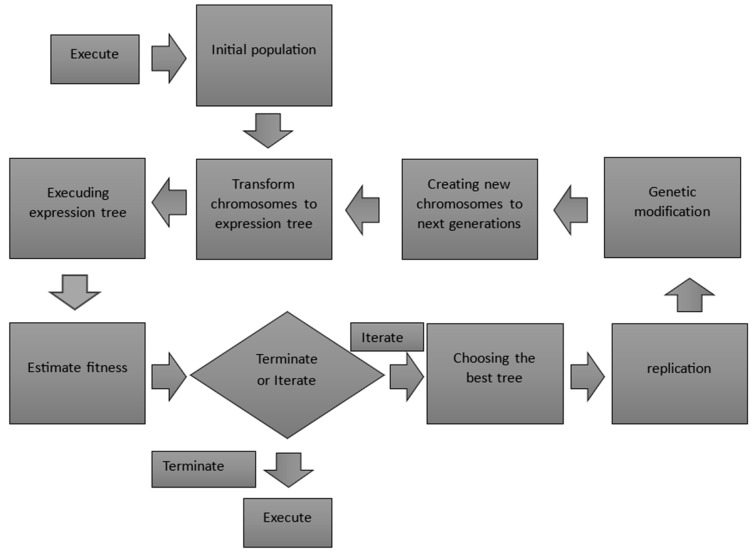
Flowchart demonstrating the model selection in gene expression programming [51].

One example of the use of gene expression algorithms in materials science is the design of materials with specific mechanical properties. Predictive models have been developed to effectively estimate the shear strength of an exterior reinforced concrete joint. The main factors considered when establishing the strength were the geometry of the beam and column, the material properties of concrete and steel, longitudinal and shear reinforcement, and axial loads on the column. Another factor in favor of using gene expression algorithms in the study of material strength was the need to improve the accuracy of shear strength predictions in reinforced concrete joints. This is because the structural failure of beam-column connections has been identified as a major cause of building collapses during earthquakes. Gene expression programming (GEP) is a widely used method capable of handling input data across various domains. GEP encodes chromosomes as linear and nonlinear sequences with varying dimensions and configurations. This characteristic of GEP enhances its efficiency compared to other methods like genetic programming and evolutionary strategies [51,52,53]. GEP functions by creating models from supplied data without being limited to a specific domain. The main difference between GEP and genetic algorithms (GA) is the type of chromosome representation. GEP includes a linear string of fixed length and a multidimensional branching framework with various potential sizes and configurations. In GAs, “chromosome” refers only to a linear string of characters of constant length. In contrast, in genetic programming (GP), “chromosomes” refers to non-linear entities of different sizes and shapes. GEP identifies the top candidates from the initial population to discover optimal solutions. Notably, increasing the number of genes and chromosomes in GEP can result in complex functions ideally matched to their outcomes. Thus, one must balance obtaining a straightforward mathematical representation with limiting the number of genes and chromosomes to achieve the desired level of accuracy. For the research, an extensive database of 256 experiments was compiled to estimate the shear capacity of exterior reinforced concrete joints subjected to cyclic loading. Among these, 156 experiments involved shear reinforcement, while 100 did not. A random subset of 256 experiments was chosen as calibration data to refine the models; the rest were used for validation. Early studies have shown that the shear strength of the joint is proportional to the compressive strength of concrete and the shear reinforcement per joint but inversely proportional to the joint aspect ratio. The gene expression algorithm used in the study showed a very high coefficient of determination R2, which was 0.94 for the unreinforced exterior joint and 0.93 for the reinforced joints. This is an increase compared to other models cited in the study from the work of [54,55,56,57]. The shear strength values were 0.55–0.88 for the unreinforced exterior joint, depending on the model used. An increase was also noted for the reinforced joints, as the range of previous models was 0.39–0.89. Figure 3 shows the flowchart demonstrating the model selection in gene expression programming.

Finally, fuzzy logic and neuro-fuzzy methods are compelling approaches that effectively characterize material properties, structures, and behaviors across various complex systems [58,59,60]. These AI-driven techniques remain vital in tasks related to classification and prediction, showcasing their enduring value and relevance in the field.

The unprecedented development of AI algorithms and computational methods allows for advanced micro and nanoscale material analyses. It enables the modeling of innovative materials based on a set of desired properties or characteristics of the environment in which a given material works.

## 4. Advanced Computational Methods for Material Modification and Property Prediction

In today’s dynamically evolving world of science and technology, integrating advanced materials science with artificial intelligence and computational methods unlocks vast opportunities. This combination allows for designing materials with unique properties. Additionally, it expands the scope of artificial intelligence for modeling, simulation, and optimizing complex systems.

### 4.1. Application of Advanced Computational Methods in the Development of Composite Materials

#### 4.1.1. Composite Shells

Composite shells are recognized for their efficient use of materials and customized material properties [61]. However, the performance of composite materials relies heavily on the precise choice of distinct factors. These factors encompass the balance between matrix and reinforcement, as well as the orientation of reinforcements, significantly influencing the mechanical characteristics of such materials. The production and optimization of composites pose a significant challenge for computational techniques. Therefore, the work attempted to optimize composite shells using artificial neural networks as a surrogate model to traditional computational methods such as the finite element method. The main problem associated with performing optimization calculations is many input parameters. The presented surrogate models appear to be a good solution. Models based on neural networks have coped with problems requiring 17 parameters with high accuracy. The performance of individual neural network surrogate models was assessed against ensembles composed of five neural networks. The objective was to ascertain which approach yields more dependable outcomes. While single deep neural network models exhibited diverse performance, ensembles of deep neural networks were effective in determining the optimal outcome without requiring extensive verification. The optimization process highlighted the efficiency of model ensembles, the significance of identifying mode shapes, and a successful balance between computational resources and optimization performance [61]. It is also important to mention that artificial intelligence methods can optimize materials and process parameters [62] in intensifying mass and heat transfer [63,64].

#### 4.1.2. Liquid Composites

The situation is similar in the process of forming liquid composites. In this case, the search for new solutions is driven by the complexity of evaluating them. Prototyping physical composites is costly, and the complexity of simulating the phenomena is a demanding computational problem. One way to accelerate calculations related to composite optimization is presented. The authors [65] emphasize that a critical aspect of optimization processes is identifying issues associated with modeling composite manufacturing processes in advance. This is important for resource utilization and minimizing the cost of producing a working model. The selection and accuracy of the appropriate quality of calculations are also essential in composite manufacturing while maintaining a low computational cost. Economical approaches that prioritize efficiency over precision can be implemented with minimal risk during the initial stages of optimization. Meanwhile, slight inaccuracies can be rectified as the process advances [65].

#### 4.1.3. Nanocomposite Membranes

The paper [66] presents the optimization of the elastic modulus for polymeric nanocomposite membranes. The mechanical characteristics of polymeric membranes play a pivotal role in ensuring their success and durability in water treatment technologies. Achieving optimal mechanical properties in membrane fabrication involves a balance among various factors such as material composition, additives, processing conditions, porosity, and other variables. Several variables that need to be optimized demand detailed experimental and computational investigations. The authors employed the design of experiments (DOEs) technique to streamline the process and reduce the number of experiments required for optimal membrane fabrication conditions. This involved using a validated framework with a computational model to predict elastic behavior. The research focused on optimizing the elastic modulus of polymeric membranes using DOE, combining computational modeling and experimental validation. The target was to determine the optimal storage modulus for polymeric nano-filled membranes at an operating temperature of 35 °C, employing a three-factor–three-level problem in the Taguchi DOE. The experiment scheme obtained from Taguchi DOE guided the prediction of the storage modulus, facilitating the fabrication of the polymeric nano-filled membrane with the optimum modulus. The predicted results indicated that the combination of polyether sulfone (PES) reinforced with 0.3 wt.% halloysite nanotubes (HNTs) yielded the optimum modulus. The fabricated PES/0.3 wt.% HNT membrane aligned well with the predicted modulus, showing a percentage error of only 3%.

### 4.2. Functionally Graded Materials

**Figure 4:**
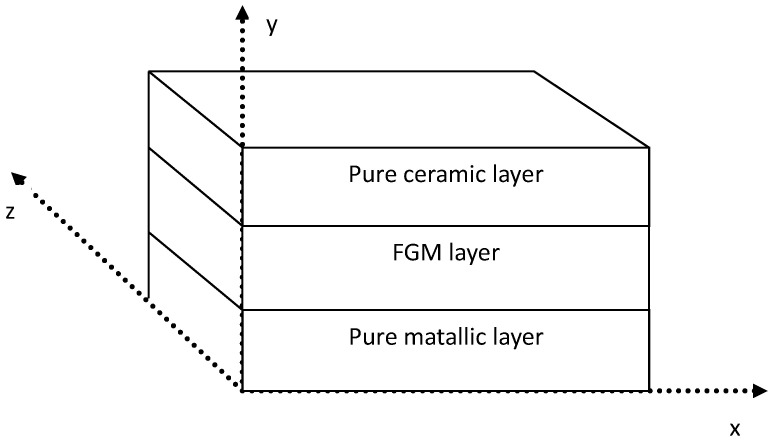
A simplified scheme of a layered composite plate with FGM.

Kazemzadeh-Parsi et al. [67] employed the model reduction technique of Proper Generalized Decomposition (PGD) for analyzing thermo-elasticity in Functionally Graded Materials (FGMs). This method eliminates the need for repetitive simulations in conducting parametric analyses, thereby overcoming the challenges posed by high dimensionality and resolving problems involving numerous parameters. In contrast, conventional grid-based approaches falter due to computational expenses. FGM proves highly valuable in space-related technologies, especially when components endure extreme thermal conditions. In this study, the authors explored material gradation in single, dual, and triple directions. Additionally, they utilized the resolution of 3D heat transfer equations and the theory of elasticity equations to achieve a precise temperature distribution and account for all shear deformations. The crucial role of parametric analysis in designing such materials guided this decision. This approach is presented in Figure 4.

Furthermore, the investigation incorporated variations in material properties across multiple directions to align with the latest advancements in the additive manufacturing of FGM materials. The inception of the PGD approach aimed to address transient issues through space-time decomposition, steering clear of conventional incremental time-stepping methodologies.

The authors of the paper [67] provide a solution that could effectively solve problems associated with many dimensions. In contrast to traditional composite materials, FGMs do not generate such high interphase stresses, which makes them less prone to failure than previous solutions [68]. This feature makes FGMs an ideal choice in harsh thermal environments. It allows for the presence of a part with a pure metallic phase in one place to provide strength and a pure ceramic phase in another place for high-temperature resistance. The authors argue that the PGD (Proper Generalized Decomposition) technique effectively eliminates the problem associated with the curse of dimensionality. It eliminates the need for repeated simulations to conduct parametric analysis. Thermoelastic analyses are critical in producing special-purpose materials, including space materials. Future research directions will focus on the possibility of calculating the exact physical properties of these materials, not only for FGM plates. The authors plan to extend their calculations to FGM panels with convex shapes. Other methods of using artificial neural network models in composite-related problems are also worth mentioning. These materials find wide applications in materials engineering in various sectors of its operation [69,70].

### 4.3. Properties and Structures Prediction of Fluoro Perovskites

**Figure 5:**
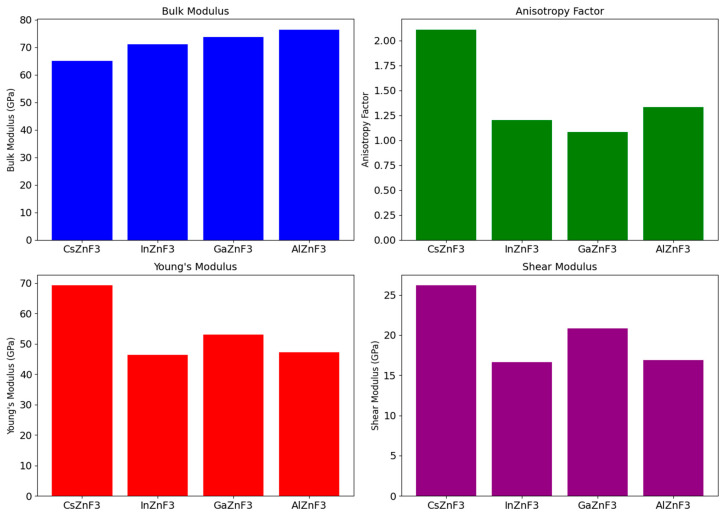
Calculated values strength parameters.

Habib et al. [71] calculated the physical properties of fluoro perovskites XZnF_3_ (X = Al, Cs, Ga, In). Using full-potential linearised augmented plane waves (FP-LAPW) enabled the determination of various material properties, including structural, elastic, electronic, and optical characteristics. To consider the influence of exchange and correlation potentials, the generalized gradient approximation (GGA) was employed during the optimization process. During the study, the values of the Poisson ratio, Cauchy pressure, and Pugh ratio were also calculated (Figure 5). The FP-LAPW approach under the generalized gradient approximation was utilized in [71,72]. The authors modeled the structural characteristics using the Birch-Murnaghan equation of state by fitting the energy-volume relationship of the crystal unit cell. The characteristics of the electrons were examined with the GGA method at specific symmetry points within the first Brillouin zone. In an actual system, electron density is distributed unevenly, unlike in an ideal free electron gas. As a result, a gradient correction to the charge density, referred to as the generalized gradient approximation, was introduced. The authors contend that GGA has been remarkably effective in improving both the efficiency and accuracy of electronic structure calculations and has emerged as the most widely used computational method in multielectron systems [71].

**Figure 6:**
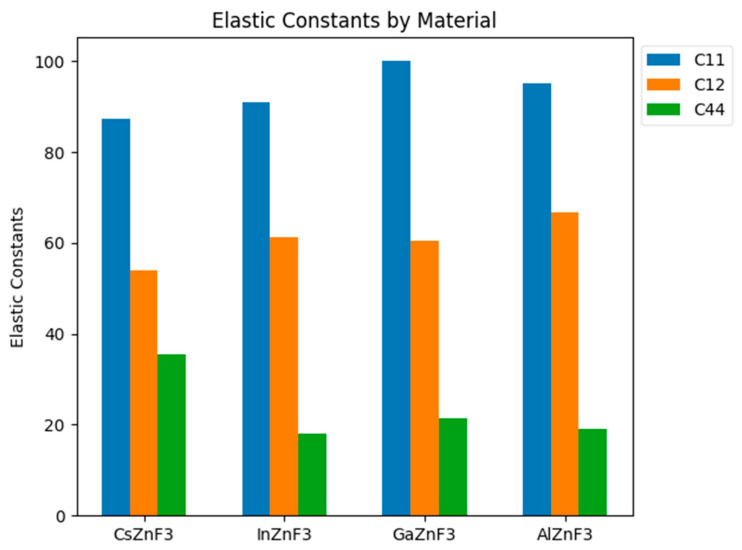
The values of elastic constants.

The text presents the importance of elastic constants (C_ij_) in describing the mechanical properties of compounds. These constants characterize the deformation of a material under stress, as well as the stability of the structure. In the case of compounds with a cubic structure, three constants are sufficient to describe the elastic properties: C_11_, C_12_, and C_44_ (Figure 6). The measured values of the constant C_44_ indicate that the compound CsZnF_3_ may be stiffer than the remaining compounds. The researchers also determined the compounds’ elastic anisotropy factor, which is essential in applied sciences and engineering. All compounds studied had plastic properties due to a Poisson’s ratio higher than 0.26 [71].

### 4.4. The Predicting of Fiber Properties

Predicting material properties and structures constitutes the basic applications of artificial intelligence in materials engineering. The paper’s authors [73] present using artificial neural networks (ANN) to predict the diameter of nanofibers produced in the electrospinning process. Metrics corresponding to various ANN architectures depend on the number of hidden neurons and layers (Table 3). The materials used in the study were polyvinyl alcohol (PVA) solution, PVA/chitosan, and PVA/aloe vera. Additionally, gelatine type A (GT)/alpha-tocopherol (α-TOC), PVA/olive oil (OO), PVA/orange essential oil (OEO), and PVA/anise oil emulsions were used. It is worth noting that attempts to predict the diameter of fibers produced in the electrospinning process have been analyzed earlier. For example, Lakshmi Narayana et al. [74] used neural networks to predict the diameter of 3D melt-electrospun polycaprolactone fibers.

The present study [73] used a multilayer neural network with a sigmoidal (logistic) activation function in the intermediate layers and a linear activation function for the output. The network underwent training with the Levenberg–Marquardt backpropagation algorithm. The dataset was partitioned into three groups: training (70%), validation (15%), and testing (15%). The network’s input layer had four neurons, one for each of the four electrospinning variables (flow rate, voltage, viscosity, and conductivity). The output layer comprised one neuron, representing the electrospun nanofiber’s diameter. The authors tested several neural network configurations, varying the number of neurons in the hidden layer and the number of hidden layers. Table 3 presents the best network configurations presented in the study. The authors confirm that the results regarding the number of neurons in the network are consistent with other studies of this type. However, they cite a study [75] in which the optimal configurations were those with a single hidden layer containing 20 neurons. Therefore, utilizing such a configuration may seem intriguing when comparing two similar networks. Nevertheless, this comparison was not provided. The optimal configuration was a network with three hidden layers of the 8-16-3 topology. This configuration had the lowest minimum mean squared error (MMSE) and the highest coefficient of determination R^2^ between the predicted and experimental fiber diameters. The results of this study show that artificial neural networks can be used to accurately predict the diameter of nanofibers produced in the electrospinning process. The findings of this study provide further evidence of the effectiveness of artificial neural networks in predicting material properties.

The artificial intelligence model presented in the study [53] for predicting the shear strength of reinforced concrete structures has been extended to include the tensile strength of steel fibers, which is often omitted in empirical models. This property strongly influences the formation of cracks in the concrete matrix and, therefore, affects the shear strength of the beam. The study and the inclusion of tensile strength as another factor improving the ability to predict shear strength is important since the study [52] has shown that the tensile strength of steel fibers can be increased by enhancing the impact resistance and shear strength of reinforced concrete beams. This is another argument in favor of its inclusion in the computational model. During the model’s development, 488 experiments were conducted to assess the shear capacity of steel fiber-reinforced concrete beams, with 190 of these experiments used for model validation. The research findings indicated that a higher span-to-depth ratio, greater effective depth, and larger aggregate diameter lead to a reduction in the beam’s shear strength. However, an increase in the compressive strength of concrete, reinforcement ratio, fiber volume, and fiber tensile strength increases the shear strength of steel fiber-reinforced concrete beams. The authors point to the accuracy and correctness of the proposed model, as the values obtained from the statistical analysis are very close to the reference values. In particular, the coefficient of determination R^2^ of 0.97 is close to the reference value of 1.00, indicating the model’s high reliability [51].

Many available AI algorithms and advanced computational models create new research areas. It defines the development prospects of these tools, and their analysis is carried out in the next section.

## 5. Emerging Strategies in Advanced Computing and AI: Exploring Future Research Directions

The observed intensive development of computation techniques, including artificial intelligence methods, provides new concepts and tools in data analysis and optimization [76]. In the traditional approach, novel materials are invented via experimentation, theory, or computation [77]. Data-driven materials science is also accessible as an alternative time-consuming and expensive experimental method [78,79,80]. The manageable data are collected in data setups, and artificial intelligence-based approaches discover new materials. A significant contribution to understanding the status, challenges, and perspectives of data-driven material science is the study by Himanen et al. [81]. This study emphasizes the critical role of selecting optimal candidate materials and the development of improved or novel materials based on machine learning methods. The authors provided some examples of such successfully synthesized novel components. They listed new molecules for organic light-emitting diodes (OLEDs) [82], polymer dielectrics for electrostatic energy storage [83], novel 12 gallides as Heusler structures [84], NiTi-based shape memory alloys with small thermal dissipation [85], lead-free piezoelectrics [86] and metallic glasses [87] and high-entropy alloys [88] for structural applications requiring hardness and corrosion resistance.

Badini et al. [36] reported the possibility of discovering new mechanisms beyond intuition in materials science. The authors drew attention to alternative AI models, e.g., reinforcement learning, to obtain an accurate model from an unidentified data domain. Such an approach allows building a model to learn a biological design strategy via several training steps. A finite element method was listed as an example to calculate mechanical properties as “reward values” for the algorithm to find materials with high fracture toughness. Fuzzy logic-based systems should also be listed here. Since this approach allows for the formalization of an empirical problem using experience rather than strict knowledge of the process, the methods run intuitively and have the potential to overcome the shortcomings of traditional approaches [89,90,91].

The basic methods for examining material properties using AI techniques are discussed by Wei et al. [92]. The summary of current machine learning methods applications in materials science, as well as the improvements that are necessary for wide-ranging applications, are also discussed in the study. Big data is accessible in materials science, allowing the development of AI methods. The machine learning workflow comprises data selection, feature engineering, modeling, and validation. Supervised, unsupervised, semi-supervised, and reinforcement learning are basic machine learning methods used in materials science. The authors also listed three essential applications of AI-based methods in materials science: material property analysis, discovering new materials, and quantum chemistry. Degradation detection, nanomaterials analysis, and molecular property prediction are listed within the first category. Discovering new materials covers structure-oriented design, element-oriented design, inverse design, and drug design. The authors emphasized that insufficient material data is challenging for ML applications in materials science. They provided an example where a small data set size of 4000 samples was sufficient to train a deep-learning model with acceptable performance [92,93]. The pivotal role of data acquired in developing a model is emphasized in [94]. The authors proposed an intelligent generation method of advanced structures. The abundant topology optimization models obtained for different parameters can serve as a base for developing outstanding models for creating deep learning datasets.

### 5.1. Utilizing Transfer Learning in Material Science

Hu et al. [95] proposed a pivotal approach for deciphering intricate interactions between materials and systems. A cutting-edge Network Representation Learning framework considered a dynamic structure and vertex attribute fusion network embedding allowed the inherent limitations of traditional Network Representation Learning methods to be overcome. Contemporary data mining approaches have also proven effective in understanding and forecasting the properties of materials. A critical aspect of the materials discovery process involves identifying which material(s) will exhibit desirable properties. Experimentation and density functional theory computations are often costly and time-intensive for many material properties. This makes it challenging to construct accurate predictive models using conventional data mining methods due to limited data availability. Therefore, enhanced predictive analytics on diverse materials datasets is presented in [96]. The authors introduced a framework for predicting material properties that utilize structural information. This framework employed a graph neural network-based architecture and incorporated deep transfer learning techniques. This significantly enhanced the model’s predictive capabilities across diverse materials, including 3D/2D, inorganic/organic, and computational/experimental data. Through evaluation in cross-property and cross-materials class scenarios across 115 datasets, the authors found that transfer learning models outperformed those trained from scratch in 104 cases, approximately 90%, with additional performance benefits for extrapolation challenges. The proposed framework held broad applicability in expediting materials discovery within materials science.

### 5.2. Ensemble Models

Zhu et al. [97] considered the issue of reactive power optimization of distribution networks. It was noticed that conventional reactive power optimization methods of distribution networks either need much calculation time or have limited accuracy. In response to the problems discussed, a novel data-driven approach was proposed in this paper. This approach simultaneously improves accuracy and reduces calculation time for reactive power optimization using ensemble learning. The K-fold cross-validation was used to train multiple sub-models, which were merged to obtain high-quality optimization results through the proposed ensemble framework. Therefore, to analyze the impact of the sub-model order on the proposed method performance, 15 cases with different rankings were set, and each case was repeated 30 times. The mean loss functions (i.e., MSE) of the test set have been shown in Table 4.

Comparing the obtained results of loss functions of Case 6, Case 13, Case 14, and Case 15, it is visible that multiple different sub-models were more conducive to improving the performance of the ensemble model than multiple identical sub-models. The authors of the work also pointed out that calculation time is one of the essential metrics used to evaluate performance. Suitable dispatching strategies should be achieved within 60 s. Therefore, the presented results proved that the authors’ proposed method’s calculation time is much lower than the traditional heuristic methods, such as the genetic algorithm. For single reactive power optimization of the modified IEEE 69-bus radial distribution network, the online time consumptions of the ensemble model, GA, CNN, MLP, LightGBM, and CBR were 0.23 s, 64.77 s, 0.08 s, 0.06 s, 0.09 s, and 4.37 s, respectively [97]. The advancement of materials science in electronic component design and optimization can be found in [98]. The authors discussed the size reduction tasks with explicit and implicit formulations. Both implicit and explicit techniques effectively control design constraints and achieve miniaturization. Implicit methods are noted for their conceptual simplicity and ease of implementation, often resulting in slightly better miniaturization rates. According to the study, explicit methods offer more precise control over design constraints. Applying ensemble learning frameworks for multiple machine learning models can be leveraged in alternative methodologies. Krzywanski et al. [99] used the automated machine learning approach (AutoML) to predict the performance of adsorption chillers, using various adsorbents in adsorption cooling and desalination systems of different construction. The DataRobot platform was a highly efficient tool for AI applications compared to traditional ANN-based applications in similar tasks [100].

### 5.3. Material Genome Technology

Artificial intelligence and machine learning are undoubtedly facilitating the development of materials engineering in biomedical engineering. Traditionally, creating new biomedical materials is based on trial and error, which can be costly and time-consuming. material genome technology (MGT) is a method that can address the issue of repeatedly creating new materials. To date, this technology has been applied to the research and development of metal, inorganic, non-metallic, polymeric, and composite biomedical materials. The MGT initiative establishes the relationship between composition, processing, microstructure, and performance to facilitate materials research and development. Establishing such a correlation can assist in advancing and improving new materials. This is necessary due to the ever-increasing demands on materials. Obtaining comprehensive data on materials can shorten the time and reduce the cost of materials development. One of the pillars of this approach is the use of well-prepared and reliable databases. The authors of [101] argue that extracting useful information from vast data is difficult. According to them, an optimal materials repository ought to accommodate extensive data storage, maintain data in a uniform manner, and offer convenient access and stored by users. Another important factor from the perspective of MGT is the use of high-throughput tools. High-throughput synthesis and characterization of materials involve the preparation and analysis of samples with varied structures or components simultaneously and in significant volumes over a short timeframe. These expedited experiments contribute to enhancing data precision and reproducibility on a larger scale. Ultimately, these processes can expedite the development of materials repositories, validate the precision of theoretical models, and explore novel materials [102]. The MGT approach also uses materials computation methods. The issues related to the relationships between materials manufacturing methods, their structure, components, and properties can be too complex for researchers to discover. Predictive models can help to identify dependencies in exceptionally complex structures. In addition, existing computational methods related to optimizing the composition of materials [103] or predicting their properties [104] will undoubtedly become valuable tools, as evidenced by their growing popularity. The authors highlight the potential of the MGT method in developing biomedical, smart, and superconducting materials. By leveraging AI methods and well-maintained large databases, this method can accelerate activities such as optimizing or searching for new biomedical materials with desired properties. The MGT method is a holistic approach that integrates experimental data, calculations, and modeling with materials science.

### 5.4. Quantum Computing

Huber et al. [105] introduced a notable strategy. They showed how creating standardized workflow interfaces that automatically calculate material properties can significantly ease interoperability and cross-verification processes. These researchers have proposed guidelines for creating reusable, code-independent process interfaces designed to compute specific material properties. This approach has been applied to eleven quantum engines for calculating a range of material properties. The authors established a unified workflow interface to optimize solid-state structures and molecular geometries to demonstrate the discussed concept. This interface is implemented across eleven quantum codes, including ABINIT, BigDFT, CASTEP, CP2K, FLEUR, Gaussian, NWChem, ORCA, Quantum ESPRESSO, SIESTA, VASP [105].

Optimization techniques and algorithms constitute a novel, useful tool for improving energy systems. Due to global aspiration to a net-zero emission economy, we are moving away from energy-intensive systems in various brands, replacing them with renewable energy sources or looking for ways to optimize conventional systems thoroughly. These activities are carried out by implementing advanced numerical methods, AI algorithms, and technologies for creating and analyzing digital twins. Examples of such applications are examined in the next section of the review paper.

## 6. Optimization Techniques and Algorithms in Energy Systems

The most complex optimization tools are artificial intelligence methods, whose effectiveness depends on available databases and computing power [106]. The simulation results showed that the time of calculation was lower than traditional heuristic methods. Additionally, the proposed attitude outperformed popular baselines such as light gradient boosting machines, convolutional neural networks and multi-layer perceptron. The simulation and optimization model of the power flow into AC-DC hybrid microgrids operating for different generation–consumption scenarios has been developed [107] using LabVIEW software. The application proposed by the authors was assembled using a multiple-input multiple-output model. This model was built using blocks containing simplified models of photovoltaic modules, wind turbines, battery arrays, and power loads. Simulations were performed using 250 W commercial solar panels, 300 W permanent magnet generator wind turbines, and 12 V batteries with a capacity of 100 Ah. The implementation of simplified models for solar panels, wind turbines, batteries, and loads, combined with binary logic to manage the microgrid operation, significantly reduced the computational cost of the simulation. Thanks to the use of average power as both an input and output variable, ease of control and reconfiguration of microgrids was achieved. The usefulness of the developed model in analyzing efficiency for different configurations of the same microgrid architecture and the possibility of extending it by integrating additional elements has been proven. A machine learning method for generating the stochastic load forecasts required by electric utilities for the evolving electrical distribution system has been introduced in [108]. The optimization analyses conducted by the authors constitute a response to the expected more significant variation in electrical load in the coming years due to the increasing popularity of electric vehicles, photovoltaics, and energy storage systems. Distribution of these solutions will vary by area and the financial capacity of customers, and if not identified early and managed by electric utilities, it could result in power dependability and protection issues.

The range of technologies used in the energy industry is extensive. They involve fuel combustion, raw material processing, waste management, or energy conversion. They are often combined systems to increase their energy efficiency. Moreover, their complexity and multi-threaded management process require advanced optimization and predictive methods to maintain long-term operation [109,110]. One innovative technology implemented in sustainable energy is fluidization. Its effectiveness is confirmed by implementations in other industrial sectors, but research on optimizing fluidized processes is ongoing. Padhi R. et al. [111] conducted experiments and numerical simulations aimed at determining the pressure within the bed, its thermal expansibility, and the fluctuations occurring within it. Following modern industry trends, multiphase fluidized beds appear to be one of the most promising devices in the fields of materials engineering, chemical, petrochemical, and pharmaceutical industries. In recent years, fluidization technology has also been utilized for drying, cooling, heating, and freezing food products [112]. A mathematical model of a fluidized bed dryer was developed in [113]. Multi-criteria optimization methods without preferences were applied, and a set of Pareto-optimal solutions was evaluated. The authors provided a detailed example of drying potato slices, demonstrating the efficiency and effectiveness of the proposed optimization approach.

The energy industry requires modeling fuel combustion processes, but their complexity makes it difficult to model them using traditional mathematical methods. Ma, YP. et al. [114] proposed a hyper-parameter self-optimized broad learning system using a sparrow search algorithm to model the thermal efficiency of a circulation fluidized bed boiler (CFBB) and the NO_x_ and SO_2_ emissions concentration. The system can be considered an extension of a more fundamental approach, as shown, e.g., in [115]. The developed broad learning system (BLS) is a novel neural network algorithm performing well in multidimensional feature learning. However, its disadvantage is that several hyper-parameters are set in a wide range, so the optimal combination is challenging. A sparrow search algorithm (SSA) to select the optimal hyper-parameters combination of the broad learning system, namely SSA-BLS, has been utilized in the paper. Ten benchmark regression datasets were applied to prove the effectiveness of SSA-BLS. Experimental results showed that the authors obtained good regression accuracy and model stability using SSA-BLS. The developed SSA-BLS algorithm has also been implemented to model the combustion process parameters of a 330 MW circulating fluidized bed boiler.

Greenhouse gas emissions were also modeled in [116]. Considering various technologies, the authors used an innovative approach based on fuzzy logic, one of the leading artificial intelligence methods, for predicting CO_2_, CO, NO_x_, and SO_2_ concentrations in coal and biomass combustion exhaust gases. The predictive simulations for different combustion environments were discussed in the paper under a wide range of operating parameters. Good agreement between the predicted emissions and experimental results has been proven in the paper, as confirmed by the maximum relative error between measured and predicted emissions under 8%. Therefore, the proposed method constitutes a quick and easy-to-run technique and a complementary tool compared to the experimental procedures. The most important effect of the conducted simulations is developing a fuzzy logic-based model of gaseous emissions from the advanced combustion of solid fuels that scientists and engineers can effectively implement to simulate and optimize coal and biomass combustion processes. Optimization of CFBB key control parameters based on artificial intelligence has also been conducted [117]. The authors of the paper noted that during the coal-fired circulating fluidized bed unit’s involvement in the power grid’s peak regulation process, the thermal automatic control system aids the operator in adjusting the mode. This system focuses on contamination control and ignores the economy, suggesting that the unit’s operating performance maintains a vast potential for deep mining. Machine learning algorithms were used for optimization. The high-dimensional and coupling-related data characteristics of CFBB put forward more demanding requirements for combustion optimization analysis and open-loop guidance operation. Therefore, the authors proposed a combustion optimization method implementing neighborhood rough set machine learning. The paper showed that the developed method first reduces the control parameters affecting multi-objective combustion optimization using the neighborhood rough set algorithm, which fully considers the correlation of each variable combination. Then, it establishes a multi-objective combustion optimization prediction model by combining the online calculation of boiler thermal efficiency. Finally, the proposed algorithm optimized the control parameter settings for the boiler combustion system. The results demonstrated that this innovative method reduced the number of control commands required for combustion optimization adjustment from 26 to 11. In [118], an optimal scheduling method for complex integrated energy systems has been proposed. The developed method implements a heuristic algorithm to maximize energy, environment, and economy indices and optimize the system operation plan. The method described in the paper uses k-means combined with box plots (Imk-means) to improve the convergence speed of the heuristic algorithm by forming its initial conditions. The authors conducted a case study to validate the developed method’s effectiveness. The results show that the proposed algorithm can decrease the running time by up to 89.29% at the most and 72.68% on average, compared with the traditional genetic algorithm. Sorption technology has the potential to provide high energy density thermal storage units with negligible losses and environmentally friendly refrigeration and desalination systems. However, significant experimental and computational advancements are necessary to unlock such green technologies’ full potential and model and improve their performance efficiently at the system scale.

The work of Scapino L. et al. [119] explores the development, application, and capabilities of neural network models to predict the performance of a sorption thermal energy storage system. Two neural network architectures were proposed to dynamically predict the state of charge, outlet temperature, and consequently, the thermal power output of a sorption storage reactor. Each neural network architecture was evaluated in 32 different configurations for the two operating modes, with a systematic training procedure used to identify the optimal configuration for each architecture and operating mode. Datasets of hydration (H) and dehydration (D) modes and the number of simulations are presented in Table 5 [119].

The authors indicated that the proposed model could accurately replicate and predict the dynamic behavior of the thermal energy storage system with mean squared error estimators below 2 × 10^−3^. Krzywanski [120] presented a general approach to optimizing the adsorption chiller’s heat exchanger system using a bio-inspired AI algorithm. The author implemented genetic algorithms and artificial neural networks for optimization analyses. The developed model was validated against the desired data on a large falling film evaporator. A broad range of operating conditions and geometric configurations were considered in the study. The results show that the total heat transfer rate of the evaporator, predicted by the model, is in good agreement with the desired data, and the maximum error is lower than +/− 3%. The modeling studies conducted in the works mentioned above, validated based on the experimental data sets, confirm the possibility of using practical artificial intelligence algorithms as advanced techniques for optimizing energy systems.

On the other side, Grabowska et al. [121] carried out interesting comparative studies of the thermal properties of coated and fixed beds of adsorption chillers. The CFD approach with conjugate heat transfer analysis allowed temperature distribution in the adsorbent bed to be determined as the essential input parameter. Besides these advanced simulation techniques, some interesting simple approaches can also be found in materials science.

The authors of [122] performed dynamic multi-objective optimization in brazier-type gasification and carbonization furnaces. With distinctive porous structure and prolonged carbon sequestration characteristics, biochar has demonstrated its potential to enhance soil fertility, mitigate carbon emissions, and augment soil carbon sequestration. Despite these benefits, the widespread adoption of biochar technology has been hindered by challenges such as the intricate structure, long transportation distances for resources, and high costs. Addressing these issues, a brazier-type gasification and carbonization furnace has been developed within [122] to conduct dry distillation and anaerobic carbonization, achieving a high carbonization rate under elevated temperature conditions.

To improve operational and maintenance efficiency, the authors present the operation of the brazier-type gasification and carbonization furnace as a dynamic multi-objective optimization problem (DMOP). Initially, an analysis of dynamic factors in the furnace’s operational process was performed, considering aspects such as equipment capacity, operating conditions, and the biomass processed by the furnace. Subsequently, biochar yield and carbon monoxide emissions were selected as dynamic objectives, and the DMOP was formally modeled. Lastly, three dynamic multi-objective evolutionary algorithms were employed to solve the optimization problem, thereby validating the efficiency of the dynamic optimization approach in the context of gasification and carbonization furnaces. Table 6 summarizes the key strategies and potential research directions, providing a structured overview of advanced computing and AI methods and their application in various research areas.

The above-discussed results confirm that AI has great potential to support modern initiatives in the energy sector.

## 7. Conclusions

The article aimed to assemble the latest literary reports on modeling, simulations, and optimization. Within the scope of the topic, research extending beyond the domain of complex systems was published, encompassing works from materials engineering and energy systems. Consequently, a decision was made to incorporate these works within the purview of the review presented.

The apparent multitude of applications of various methods, techniques, and algorithms confirms that we possess an enormous base of tools capable of accelerating research progress. The work demonstrates the utilization of well-established algorithms, their various modifications, and those whose potential we are just beginning to explore. The abundance of these tools and their usage across diverse, often uncorrelated domains suggests a new research direction: the necessity of systematizing knowledge regarding which modern algorithms perform best in specific fields. Creating a series of literature reviews on the progress, development, available algorithm modifications, and their application’s effectiveness in a given domain would facilitate further work with advanced computational methods.

The new strategies and described cases indicate the necessity of building large and widely accessible material databases, as data fuels modern algorithms. Therefore, it seems reasonable to conclude that the broadly understood Internet of Things should permanently establish itself in every branch of engineering. Undoubtedly, the creation of databases will not only facilitate the design and prototyping of new materials but will also contribute to better environmental protection by reducing the amount of wasted resources on failed experiments. In this case, the success of design processes may depend on adopted methodologies such as material genome technology.

Despite the global trend towards utilizing artificial intelligence algorithms and machine learning, it is crucial not to overlook well-established computational methods such as numerical simulations. These methods remain effective, and when combined with the aforementioned artificial intelligence, they can continue to provide high-quality data to numerous research teams.

The findings of this study, clearly indicate the need for integrating traditional computational methods with artificial intelligence algorithms. This integration opens new avenues for development, enabling the acquisition of high-quality data and supporting research teams in various domains of science and technology. These observations point to the potential for significant acceleration of scientific and technological progress, facilitating better understanding and more effective utilization of advanced computational methods.

Finally, progress in modeling, simulations, and optimization of complex systems should also aim to solve ecological and economic aspects. This is very important due to increasingly restrictive legal and environmental regulations and the need for rational waste management.

## Figures and Tables

**Table 1:** Experimental results of a core network traffic dataset in European cities [30].

	Mean Squared Error	Root Mean Squared Error	Mean Absolute Error	Mean Absolute Percentage Error	Moving Average
Sparrow Search Algorithm Broad Learning System (SSA-BLS)	0.0159	0.1261	0.0937	0.0294	97.0572%
Stochastic Configuration Networks (SCN)	0.0155	0.1244	0.0935	0.0296	97.0434%
Extreme Learning Machine (ELM)	0.1395	0.3686	0.2711	0.0780	92.1975%
Long Short-Term Memory (LSTM)	0.0781	0.2502	0.1885	0.0518	94.8246%

**Table 2:** Comparison of Sintering Temperature prediction methods to the combined GRP-LSTM-GAN method.

Method	Short Overview of Method Characteristics
A thermal model of a rotary kiln [1]	Predicts heat transfer, temperature distribution in bed and refractory wall, considers dynamic interactions in kiln environment.
Modeling of a soft temperature field sensor in a rotary kiln [41]	Predicts temperature distribution using computational fluid dynamics and multilayer perceptrons, utilizes air temperature, speed, and material mass flow as input data.
Dynamic feature method of a series of blurry flame images [42]	Accurately segments flame regions from blurry images, extracts luminous and dynamic features to address rapid temperature fluctuations.
Generative Adversarial Networks (GAN) [43]	Captures data distributions through unsupervised learning, generates realistic synthetic data, applied in image analysis, video processing, and language comprehension.
Global Recurrence Plot (GRP) [44]	Visualizes recurring patterns in data, enhances understanding of data relationships in signal processing and time-frequency analysis.
Long Short-Term Memory (LSTM) [45]	Integrates mechanisms for long-term information retention in machine learning applications, overcomes training difficulties in sequential data analysis.
GRP-LSTM-GAN method [40]	Transforms time series into images, maximizing the utilization of temporal data to enhance temperature prediction using LSTM-enhanced GAN models.

**Table 3:** Matrics of ANN used to predict the diameter of nanofibers [73].

		One Hidden Layer		Two Hidden Layer		Three Hidden Layer	
Number of neurons		8	10	8-16	8-20	8-16-3	8-16-5
R^2^	Training	0.97	0.97	0.98	0.98	0.99	0.98
	Test	0.82	0.80	0.86	0.88	0.92	0.93
	Validation	0.96	0.97	0.98	0.97	0.97	0.96
MMSE	Training	0.03	0.03	0.02	0.02	0.02	0.02
	Test	0.08	0.10	0.06	0.09	0.04	0.03
	Validation	0.04	0.03	0.03	0.03	0.03	0.03

**Table 4:** Results of ensemble models with different orders [97].

No. of Case	Order of Sub-Models	MSE (p.u.)
1	CNN, MLP, LightGBM	0.0185
2	CNN, LightGBM, MLP	0.0203
3	MLP, CNN, LightGBM	0.0191
4	MLP, LightGBM, CNN	0.0194
5	LightGBM, CNN, MLP	0.0180
6	LightGBM, MLP, CNN	0.0157
7	CNN, LightGBM, LightGBM	0.0177
8	CNN, MLP, MLP	0.0191
9	MLP, CNN, CNN	0.0175
10	MLP, LightGBM, LightGBM	0.0176
11	LightGBM, MLP, MLP	0.0191
12	LightGBM, CNN, CNN	0.0168
13	CNN, CNN, CNN	0.0179
14	MLP, MLP, MLP	0.0181
15	LightGBM, LightGBM, LightGBM	0.0188

CNN—convolutional neural network, MLP—multi-layer perceptron LightGBM—light gradient boosting machine.

**Table 5:** Datasets of hydration (H) and dehydration (D) modes and the number of simulations.

Mode	Dataset	Parameters	Min	Step	Max	No. of Simulations
Hydration	Training	T_in_	10	5	45	64
		C_in_	0.300	0.05	0.65	
	Validation	T_in_	12.5	5	42.5	49
		C_in_	0.325	0.05	0.62	
Dehydration	Training	T_in_	70	10	150	72
		C_in_	0.20	0.05	0.55	
	Validation	T_in_	75	10	145	56
		C_in_	0.225	0.05	0.525	

T_in_—temperature at the inlet [°C], C_in_—sorbate concentration at the inlet [mol/m^3^].

**Table 6:** Key strategies and potential research directions in advanced computing and AI methods.

Author and Year	Reference	Strategy	Potential Research Direction
Gnatowski et al., 2022	[123]	Computer simulations of injection processes	Improvement of manufacturing process quality
Qiu et al., 2023	[101]	Materials Genome Technology in biomedical materials	Rapid prediction and optimization of material properties
Badini et al., 2023	[36]	AI in materials design	Discovery of materials with high fracture toughness
Ward et al., 2016	[37]	Machine learning framework for predicting properties of inorganic materials	Enhancing predictive accuracy through dataset partitioning
Goswami et al., 2023	[124]	AI in Material Engineering	Acceleration of drug development
Surmiak et al., 2020	[102]	High-throughput characterization of perovskite solar cells for rapid combinatorial screening	Developing fully automated, high-throughput characterization techniques for perovskite solar cells to expedite the research and development process.
Wang et al., 2021	[103]	Using hierarchical structures at multiple scales to simultaneously enhance the strength and plasticity of steel	Application of high-throughput methods and big data for rapid material design. Solutions for industrial-scale steel manufacturing with hierarchical structures, including advanced technologies like additive manufacturing.
Kheiri et al., 2020	[104]	COMSOL Multiphysics simulations	Optimization and prediction of material properties
Zhu et al., 2022	[97]	Data-driven approach for reactive power optimization	Improvement of calculation time and accuracy in power optimization
Gupta et al., 2024	[96]	Deep transfer learning for predictive analytics on materials datasets	Expediting materials discovery across diverse data
Krzywanski et al., 2023	[76]	Technological and modeling progress in green engineering	Sustainable development and energy materials engineering
Krzywanski et al., 2010	[77]	Modelling of solid fuel combustion	Emissions reduction in fluidized bed boilers
Gnatowski et al., 2022; Kijo-Kleczkowska et al., 2023; Grabowska et al., 2021	[78,79,80]	Thermomechanical properties analysis and waste combustion research	Mercury emissions and heat transfer adsorption bed optimization
Himanen et al., 2019	[81]	Data-driven materials science	Development of novel materials via AI-based methods
Gómez-Bombarelli et al., 2016; Mannodi-Kanakkithodi et al., 2016; Oliynyk et al., 2016; Xue et al., 2016; Ren et al., 2024; Wen et al., 2019	[82,83,84,85,86,87]	Machine learning for material discovery	Synthesis of novel components for various applications
Wei et al., 2019	[92]	Machine learning in materials science	Broadening applications of AI in material property analysis
Raccuglia et al., 2016	[93]	Use of failed experiments in ML-assisted materials discovery	Efficient data utilization for materials discovery
Li and Liu, 2022	[38]	Predictive strategy based on time-series data analysis	Investment and decision-making in market trends
Hu et al., 2022	[95]	Network Representation Learning for materials and systems	Deciphering complex interactions within materials
Roussel et al., 2022	[125]	Sensor fusion for occupancy estimation	Enhancement of predictive performance in complex environments
Pietrenko-Dabrowska et al., 2022	[98]	Optimization-based circuit miniaturization	Control of design constraints and miniaturization
Vivekanandan et al., 2023	[99]	Reinforcement learning for job scheduling	Resource allocation and efficiency in manufacturing
Algarni & Sheldon, 2023	[126]	Recommendation systems for course selection	Energy saving and efficiency in education
Wang et al., 2021	[94]	Innovative structure generation	Creation of deep learning datasets from topology optimization
Aamir et al., 2020	[89]	Fuzzy logic in multi-hole drilling optimization	Process parameter optimization in manufacturing
Krzywanski et al., 2020	[90]	Fuzzy logic in fluidized bed jet milling	Optimization of mass and particle size
Otwinowski et al., 2022	[91]	An AI fuzzy logic-based system for air classification	Improvement of classification processes
Gaspar-Cunha et al., 2022	[127]	Optimization in polymer processing	Application of AI approaches in polymer technologies
Ongar et al., 2023	[128]	3D mathematical modeling in boiler design	Reduction in NOx emissions in boiler operation

## Data Availability

Not applicable.
